# APRIL-producing eosinophils are involved in gastric MALT lymphomagenesis induced by *Helicobacter sp* infection

**DOI:** 10.1038/s41598-020-71792-3

**Published:** 2020-09-09

**Authors:** Alice Blosse, Sara Peru, Michael Levy, Benoit Marteyn, Pauline Floch, Elodie Sifré, Alban Giese, Martine Prochazkova-Carlotti, Lamia Azzi Martin, Pierre Dubus, Francis Mégraud, Agnès Ruskone Fournestraux, Bettina Fabiani, Christiane Copie Bergman, Cyrielle Robe, Michael Hahne, Bertrand Huard, Philippe Lehours

**Affiliations:** 1 Univ. Bordeaux, INSERM, BaRITOn, U1053, 33000 Bordeaux, France; 2 EC2M3: Department of Academic Research (EA7375), Université Paris Est Créteil (UPEC) – Val de Marne, 94000 Créteil, France; 3 Department of Gastroenterology, Henri Mondor Hospital, APHP, 94000 Créteil, France; 4 Institut Pasteur, Unité de Pathogénie Microbienne Moléculaire, 28 rue du Dr Roux, 75724 Paris Cedex 15, France; 5 INSERM Unité 1202, 28 rue du Dr Roux, 75724 Paris Cedex 15, France; 6 French National Reference Center for Campylobacters and Helicobacters, Hôpital Pellegrin, 33076 Bordeaux, France; 7 Department of Gastroenterology, Groupe d’Etude des Lymphomes Digestifs (GELD), Hôpital Saint Antoine, AP-HP, 75012 Paris, France; 8 Department of Pathology, Hôpital Saint Antoine, APHP, 75012 Paris, France; 9 Department of Pathology, Henri Mondor Hospital, APHP, INSERM U955, Equipe 9, Université Paris-Est, 94000 Créteil, France; 10 INSERM U955, Equipe 9, Mondor Institute of Biomedical Research, 94000 Créteil, France; 11 IGMM, Univ Montpellier, CNRS, Montpellier, France; 12 Institute for Advanced Biosciences, INSERM U1209, CNRS UMR 5309, University Grenoble-Alpes, 38700 La Tronche, France; 13 CHU de Bordeaux, 33000 Bordeaux, France

**Keywords:** Bacterial pathogenesis, Gastric cancer

## Abstract

The roles of the inflammatory response and production of a proliferation-inducing ligand (APRIL) cytokine in gastric mucosa-associated lymphoid tissue (MALT) lymphomagenesis induced by *Helicobacter* species infection are not clearly understood. We characterized the gastric mucosal inflammatory response associated with gastric MALT lymphoma (GML) and identified APRIL-producing cells in two model systems: an APRIL transgenic mouse model of GML induced by *Helicobacter* infection (Tg-hAPRIL) and human gastric biopsy samples from *Helicobacter pylori*-infected GML patients. In the mouse model, polarization of T helper 1 (*tbet*), T helper 2 (*gata3*), and regulatory T cell (*foxp3*) responses was evaluated by quantitative PCR. In humans, a significant increase in *april* gene expression was observed in GML compared to gastritis. APRIL-producing cells were eosinophilic polynuclear cells located within lymphoid infiltrates, and tumoral B lymphocytes were targeted by APRIL. Together, the results of this study demonstrate that the Treg-balanced inflammatory environment is important for gastric lymphomagenesis induced by *Helicobacter* species, and suggest the pro-tumorigenic potential of APRIL-producing eosinophils.

## Introduction

The bacterium *Helicobacter pylori* induces chronic inflammation called gastritis within the gastric mucosa in humans. Gastritis is often asymptomatic but can progress to more serious pathologies such as gastric and duodenal ulcer, adenocarcinoma and, in approximately 0.1% of infected patients, gastric mucosa-associated lymphoid tissue (MALT) lymphoma (GML)^1,2^.

In GML, chronic antigenic stimulation by *H. pylori* on the gastric mucosa induces the formation of B-cell monoclonal infiltrates with a marginal zone immunophenotype^1,3^. Reactive T lymphocytes (predominantly CD4^+^) are found within the infiltrates. These CD4^+^ cells allow the activation and proliferation of neoplastic B cells via CD40L-CD40 co-stimulation and the secretion of T helper type 2 cytokines such as interleukin 4^3,4^. Some of these T cells are regulatory cells (LTregs) that regulate local T-cell responses induced in the lymphoid follicles^5,6^. The presence of LTregs as promoters of B-cell proliferation, either by directly stimulating B cells or suppressing T cells, was suggested by Craig et al.^7^ LTregs may also participate in the persistent chronic inflammation of the gastric mucosa due to *H. pylori*, as suggested by Laur et al.^5^ rendering the antigenic stimulation process mandatory for B-cell proliferation. Compared to non-responders, GML patients with high tumor LTreg infiltration show a better response to antibiotics, suggesting that LTregs play a role in the regression mechanisms of GML^8,9^.

A proliferation-inducing ligand (APRIL) cytokine is a member of the tumor necrosis factor (TNF) family. APRIL shares protein homology with other members such as the B-cell activating factor (BAFF). APRIL and BAFF are synthesized as transmembrane type II proteins with a C-terminal domain, characteristic of members of the TNF family. APRIL is produced by cleavage into a soluble protein by a furin-like protease intracellularly before secretion. It is expressed by many cells of the immune system cells such as neutrophils, eosinophils, and macrophages^10–12^.

The association of APRIL with carcinogenesis has long been suspected^13^. Accordingly, its role in cancer and lymphoma has been analyzed in several studies^12,14–16^, but there have been few studies on the role of APRIL in GML^17,18^. Tumor-infiltrating macrophages are a major source of APRIL in GML^17^; the production of APRIL is induced by *H. pylori* antigens and *H. pylori*-specific T cells^17^.

Since it was determined that *H. pylori* plays a role in the development of GML, many animal models have been developed to understand more fully the pathophysiology of GML. An alternative model of lymphomagenesis was used in our laboratory, based on *Helicobacter sp* infection of transgenic C57BL6 mice with the human form of the APRIL cytokine (Tg-hAPRIL)^19^.

We characterized the inflammatory response in the stomach of Tg-hAPRIL mice, a novel animal model of gastric lymphomagenesis, and validated these results in the gastric biopsies of GML patients. We evaluated the inflammatory response in gastric mucosa, verified the deregulation of APRIL in human GML, and identified cytokine-producing and target cells. Our results demonstrate that a Treg-balanced inflammatory environment is important for gastric lymphomagenesis induced by *Helicobacter* species, and suggest the pro-tumorigenic potential of APRIL-producing eosinophils.

## Materials and methods

### Murine material

Murine material from a previous mouse Tg-hAPRIL experiment was obtained as follows. The stomachs of WT or Tg-hAPRIL mice, uninfected (n = 9 each) or infected with *H. pylori* (n = 16 for WT and n = 14 for Tg-hAPRIL) or *H. felis* (n = 13 and n = 10, respectively), had been recovered 18 months post-infection as described by Floch et al*.*^19^ One part of the stomach was formalin-fixed paraffin-embedded (FFPE), and another part was ground to recover leukocyte cells. A fraction of these cells had been cultured in RPMI medium overnight. Leukocytes and leukocyte supernatants (frozen at − 80 °C after centrifugation) were available for our study^19^.

### Patient selection

Eleven cases of primary GML (seven men, four women, mean age: 59.9 years) associated with *H. pylori* infection without t(11;18) (API2-MALT1) translocation were included in the present study. All GML patients were *H. pylori*-positive: nine were positive by histology, one by PCR detection on gastric biopsy only, and one by serology only. All patients had stage I gastritis according to the Ann Arbor staging system, and were treated with the typical antibiotic regimen for *H. pylori* eradication^20^, which was followed by lymphoma regression.

The diagnosis of GML was based on histological analysis of gastric biopsies by an expert hematopathologist. GML was diagnosed according to the World Health Organization classification. The presence of the t(11;18) translocation was determined by amplification and sequencing of the API2-MALT1 fusion transcript according to a previously described method until 2012, and thereafter by interphase fluorescent in situ hybridization (FISH) using the MALT1 break apart probe as previously described^21^. *H. pylori* infection was assessed by modified Giemsa-stained tissue sections and/or immunohistochemistry (IHC) with an anti-*H. pylori* antibody on FFPE tissue sections of gastric biopsies. In addition, a quantitative polymerase chain reaction (qPCR) assay was performed on frozen gastric biopsies as previously described^22^. The control group consisted of 16 gastric biopsies from patients referred for gastric endoscopy for gastric dyspepsia (n = 14), routine follow-up in the context of atrophic gastritis (n = 1), or exploration of anemia (n = 1) (six men, 10 women, mean age: 62.7 years + /− 12.1). We also used gastric biopsy blocks (sent from Saint Antoine Hospital, Paris, France) from these GML patients. The results are shown for two patients (40-year-old male, 62-year-old female), and are representative of the results obtained for the entire GML study population.

### RNA extraction

The leukocyte RNAs of WT or Tg-hAPRIL mice, uninfected (n = 5 each) or infected with *H. pylori* (n = 10 for WT and n = 8 for Tg-hAPRIL) or *H. felis* (n = 11 and n = 10, respectively), were extracted according to a standard phenol–chloroform protocol. TRIzol Reagent (Life Technologies, Carlsbad, CA, USA) was used before adding chloroform (ratio 1:5). Centrifugation at 12,000 g for 15 min allowed recovery of the aqueous phase containing the RNAs. The addition of isopropanol (ratio 1:1) followed by an additional 20 min 12,000 g centrifugation was used to concentrate the RNA in a pellet. The pellet was washed with 75% ethanol before resuspending in water. RNA quantification was performed using a spectrophotometer (BMG Labtech, Ortenberg, Germany) at 260 nm. The 260/280 nm ratio is a reflection of the quality and protein contamination in a sample. Total RNAs were also extracted from frozen gastric samples from GML individuals and the control group using TRIzol reagent according to the manufacturer’s instructions, and were quantified using the NanoDrop. The QuantiTect Reverse Transcription Kit (Qiagen, Courtaboeuf, France) was used to reverse transcribe all murine RNAs extracted from leukocyte pellets and RNAs extracted from humans. A total of 250 ng human RNA and 500 ng murine RNA were used to generate cDNA. The first step of genomic DNA removal was performed according to the supplier’s recommendations (2 min at 42 °C), followed by reverse transcription to cDNA for 30 min at 42 °C and then for 3 min at 95 °C. The generated cDNAs were stored at − 20 °C.

### qPCR

qPCR was performed with the CFX96 thermocycler (Bio-Rad, Marnes la Coquette, France) using a qPCR platform (from SFR TransBioMed, Bordeaux, France), and 96-well plates (Bio-Rad). SYBR Premix Ex Taq™ (Tli RNaseH Plus) reaction mixture (Takara Bio Inc., Shiga, Japan) was used (as recommended by the supplier) with 0.2 μM specific primers and 10 ng murine or 5 ng human cDNA. Each target was tested in duplicate for all samples. Commercial primer pairs (RT^2^ qPCR Primer Assay, Qiagen) or synthesized primer pairs (Eurofins Mwg Biotech, Courtaboeuf, France) were used to evaluate the relative expression level of human *april* (PPH01047C), human and murine *tbet* genes (PPH00396A, PPM03727A, respectively), human and murine *gata3* (PPH02143A, PPM05199A, respectively), and human and murine *foxp3* (PPH00029C, PPM05497F, respectively) compared to murine *hprt1* (Eurofins) and murine *gapdh* (Eurofins) or human *hprt1* (Eurofins) and human *gusb* (PPH01096H). Gene expression was compared by obtaining delta threshold cycle values (ΔCt) and normalizing to the expression of the two housekeeping genes. Relative gene expression values were calculated as 2-ΔΔCt according to the method described by Livak and Schmittgen^23^. Cycle threshold (Ct) values above 35 were considered non-specific and not used for the analysis.

### IHC

Serial sections (3 μm thick) were prepared, deposited on glass slides, and deparaffinized at 60 °C. After rehydration of the slides by successive toluene and alcohol baths (100 °C and 95 °C, respectively), the antigenic sites were unmasked. This unmasking was performed with primary antibody, using homemade buffers citrate (pH 6) or Tris EDTA (pH 9), for 15 or 30 min at 100 °C, and then 30 min at room temperature. Hydrogen peroxide (3%) (Sigma-Aldrich, Saint Quentin Fallavier, France) was used for 5 min to inactivate the endogenous peroxidases. Specific sites were blocked with horse serum solution. An adjustment was made to determine the optimal dilutions of each primary antibody (Table S1). The primary antibodies were labeled with secondary antibodies (Table S1) and the labels were revealed using Liquid DAB + Substrate Chromogen (Dako) peroxidase substrate followed by hematoxylin counterstaining (VWR International, Fontenay-Sous-Bois, France). The slides were subsequently dehydrated by successive alcohol and toluene baths (95 °C and 100 °C, respectively) to mount them between the slide and cover with the Eukitt mounting medium (VWR). The slides were scanned using the MIRAX SCAN scanner (3DHISTECH Ltd, Budapest, Hungary) and the images were read using Pannoramic Viewer software (3DHISTECH Ltd).

### Immunofluorescence

Serial sections (3 or 10 μm thick) were prepared, deposited on glass slides, deparaffinized, and rehydrated. To reveal primary antibody binding, fluorochrome-coupled secondary antibodies were used (Table S1). Slides were mounted between the slide and coverslip with ProLong™ Gold Antifade reagent or ProLong™ Diamond Antifade Mountant with DAPI (Invitrogen), and stored at 4 °C. The images were captured with the Nikon TE50i epi-fluorescence microscope equipped with a 40 × immersion objective and DS-Qi1Mc camera (Nikon, Champigny sur Marne, France), and then processed with NIS-BR software version 3.2 (Nikon) or with the Leica Microsystems SP5 confocal microscope equipped with a 63 × immersion objective and camera (Leica Microsystems, Wetzlar, Germany). Then the images were processed with Leica Application Suite Advanced Fluorescence software and ImageJ image analysis software version 1.48 (Java).

### Purification of human neutrophils and eosinophils

Human peripheral blood was collected by venipuncture in tubes containing sodium citrate (3.8% final) as the anticoagulant. The neutrophils and eosinophilic polynuclear cells were purified with the MACSxpress Whole Blood Neutrophil Isolation Kit and the MACSxpress Whole Blood Eosinophil Isolation Kit (Miltenyi Biotec, Bergisch Gladbach, Germany) according to the supplier’s recommendations, respectively. Son et al*.*^24^ reported that the MACSxpress method consistently allowed high-purity isolation of eosinophils (95.0 ± 1.7%) and neutrophils (94.2 ± 10.1%). Then the cells were deposited on slides by cytocentrifugation (150×*g*, 4 min) and fixed in paraformaldehyde (4%). The fixed slides were stored at 4 °C until use. The purity of the purified cells was verified by May-Grunwald staining after cytocentrifugation.

### Statistical analysis

To evaluate the significance of the results, statistical tests were performed using GraphPad Prism 6.0 software (GraphPad Software, Inc., La Jolla, CA, USA). The Mann–Whitney test was used as a non-parametric test to compare the distributions of two unmatched groups. *p* < 0.05 was considered statistically significant.

### Ethical issues

All methods were carried out in accordance with relevant guidelines and regulations. All experimental protocols were approved by a named institutional and/or licensing committee. Patients were recruited from the Gastroenterology Department’s local database at Henri Mondor Hospital and/or the standardized hospital inpatient diagnostic database from 2001 to 2016. The study was approved by the Institutional Ethics Committee (Comité de protection des personnes, Protocol No. 15071-ID RCB:2015-A00342-47). Written informed consent of non-opposition to the use of biological material for study purposes was obtained from all patients. Human peripheral blood was also collected by venous puncture from healthy patients at the Bordeaux Blood Bank (Authorization No. 14PLER006). All participants gave their consent in accordance with the Helsinki Declaration of Principles.

*Helicobacter*-free C57BL6 mice were obtained from Charles River Laboratories (L’Arbresle, France). APRIL transgenic (Tg) C57BL6 mice were originally provided by Dr. Michael Hahne (Université de Montpellier, Institut de Génétique Moléculaire de Montpellier (IGMM)-UMR5535). All experiments were performed in specific pathogen-free animal facilities at the University of Bordeaux. Animal experiments were performed according to EU recommendations (European Directive 2010/63/EU) for animal experimentation. This study was evaluated by the local ethics committee of the University of Bordeaux and conformed to the French Ministry of Agriculture Guidelines on Animal Care and the French Committee of Genetic Engineering, with respect to the principle of the 3Rs (Replace, Reduce, Refined) (Approval No. 50120143-A). Only female neonates were used for experiments.

## Results

### Characterization of the gastric mucosal inflammatory response in a mouse model of gastric lymphomagenesis

**Figure 1:**
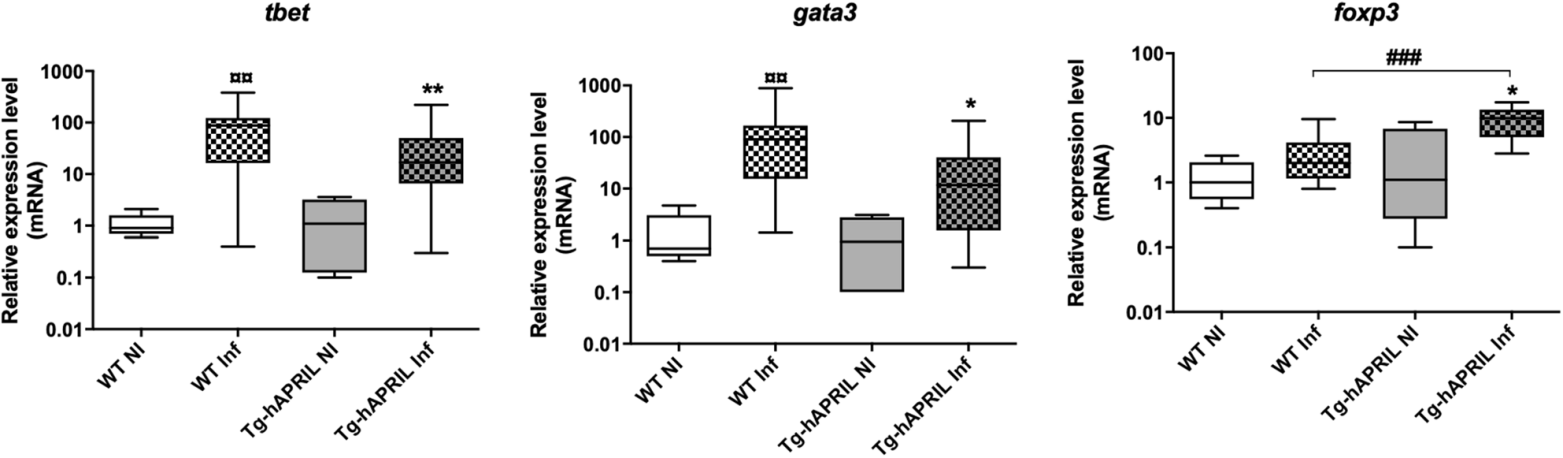
Polarization of gastric inflammatory response in WT and Tg-hAPRIL mice infected with *Helicobacter sp*. Relative expression of *tbet*, *gata3*, and *foxp3* genes in relation to NI control condition. Quantification by qPCR in leukocyte pellets according to genotype (WT or Tg-hAPRIL) and infectious status [non-infected (NI) (n = 5), infected (Inf) mice (n = 21 and n = 18, respectively)]. Boxed graphical representations, with the box representing 50% of values around the median (horizontal bar) and extremity segments representing extreme values, *(vs*.* Tg-hAPRIL NI): *p* < 0.05, ¤¤(vs. WT NI) or **(vs. Tg-hAPRIL NI): *p* < 0.01 and ###: *p* < 0.0001; Mann–Whitney test.

The relative expression levels of *tbet* (Th1), *gata3* (Th2), and *foxp3* (LTreg) genes in the leukocyte pellets were evaluated to characterize the polarization state of the infiltrating leukocytes at the GML stage in the mouse model Tg-hAPRIL. By qPCR, a significant increase in *tbet* and *gata3* gene expression was observed in infected WT and Tg-hAPRIL mice (*H. pylori* or *H. felis*). By comparing the relative expression levels of *tbet* with *gata3*, no significant difference was found in infected WT mice or Tg-hAPRIL mice, indicating the presence of a balanced Th1 and Th2 response within the gastric microenvironment (Fig. 1). In infected Tg-hAPRIL mice (*H. pylori* or *H. felis*), a significant increase in the relative expression of *foxp3* transcripts was also found, unlike in infected WT mice (Fig. 1), indicating the existence of a regulatory response in *Helicobacter*-infected Tg-hAPRIL at the GML stage. By comparing the relative expression levels of one gene with the others in this mouse model of GML, no significant difference was found, suggesting the presence of balanced Th1, Th2 and Treg responses within the gastric microenvironment at the GML stage.

### Characterization of gastric mucosal inflammatory response in *H. pylori*-infected GML patients

**Figure 2:**
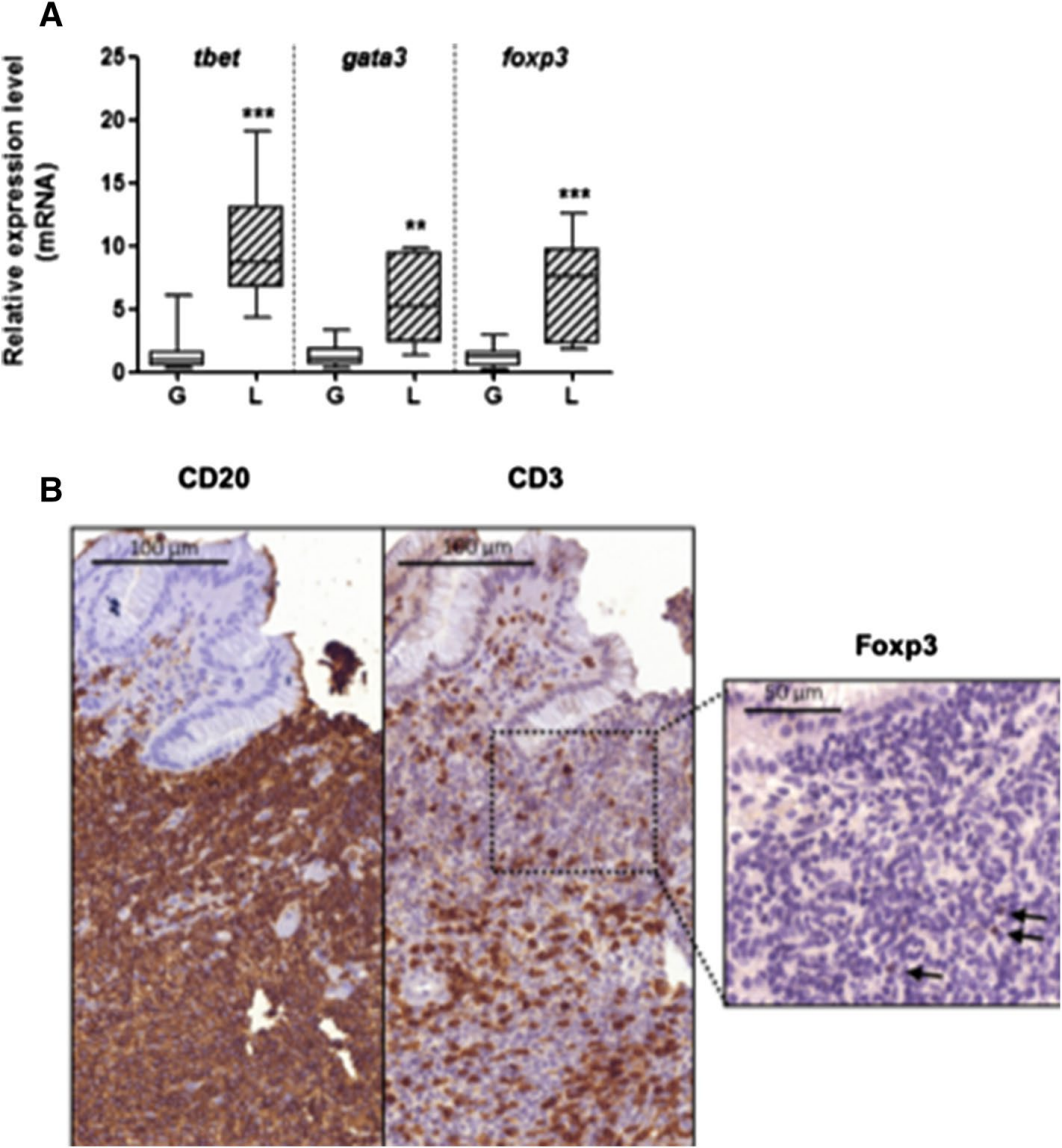
Polarization of gastric inflammatory response in patients with gastric MALT lymphoma. (**A**) Relative expression of *tbet*, *gata3* and *foxp3* genes relative to the gastritis control condition. Quantification by qPCR within RNA extracted from gastric biopsies of GML (L) patients (n = 11) or gastritis (G) control population (n = 16), all infected with *H. pylori*. Boxed graphical representations, with the box representing 50% of values around the median (horizontal bar) and extremity segments representing extreme values, **(vs. Gastritis): *p* < 0.01, ***(vs. Gastritis): *p* < 0.001; Mann–Whitney test. (**B**) Example of B lymphocyte (CD20+), T lymphocyte (CD3+) and T regulatory cells (Foxp 3+, black arrows) staining within lymphoid infiltrate by IHC on sections of human gastric biopsies from GML patients.

The evaluation of the relative expression levels of the *tbet*,* gata3*, and *foxp3* gene transcripts in RNA extracts from gastric biopsies of *H. pylori-*infected GML patients allowed characterization at the gastric level of the polarization of the inflammatory response of these patients, as compared to the gastritis control population. By qPCR, a significant increase in *tbet, gata3*, and *foxp3* gene expression was observed, confirming the presence of Th1, Th2, and regulatory responses in the human gastric microenvironment of GML patients (Fig. 2A) with no significant difference found between the relative gene expression levels. In addition, IHC detection of the CD20 surface marker of B lymphocytes as well as the CD3 and Foxp3 markers for T lymphocytes and LTregs confirmed the expected presence of lymphoid infiltrates composed mainly of B lymphocytes with some infiltrating T cells, among which LTregs were found (Fig. 2B).

### Gastric characterization of APRIL expression in *H. pylori*-infected GML patients

**Figure 3:**
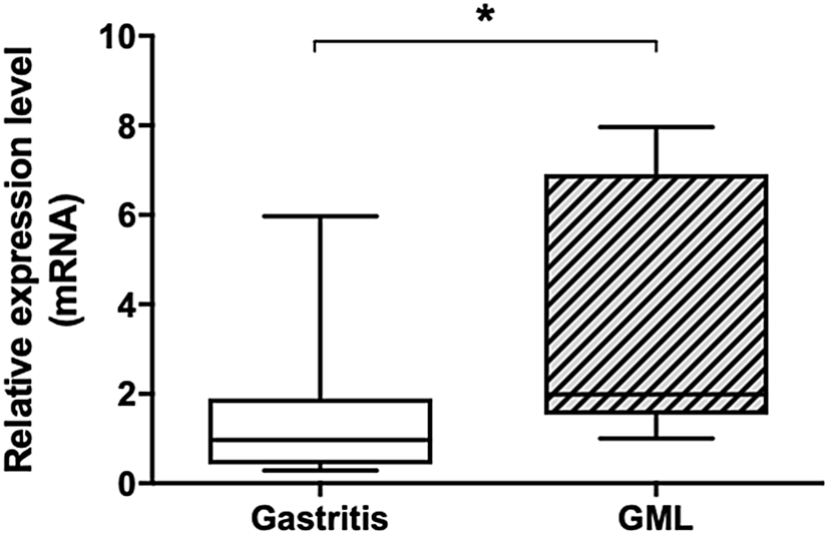
Quantification of the relative expression of the cytokine APRIL in patients with gastric MALT lymphoma. Relative expression of the *april* gene with respect to the gastritis control condition. Quantification by qPCR within RNA extracted from gastric biopsies of GML (L) (n = 11) or gastritis (G) patients (n = 16), all infected with *H. pylori.* Graphic representations are box plots, with the box representing 50% of values around the median (horizontal line) and the whiskers representing the minimum and maximum of all the data *(vs. Gastritis): *p* < 0.05.

To verify the dysregulation of APRIL in human GML, the relative expression levels of the *april* gene transcripts within RNA extracts from gastric biopsies from *H. pylori*-infected GML patients were evaluated. A significant increase in expression of the *april* gene, compared to gastritis, confirmed the presence of deregulation of this cytokine at the GML stage within the human gastric microenvironment (Fig. 3).

### Identification of producer cells and target cells of the APRIL cytokine

**Figure 4:**
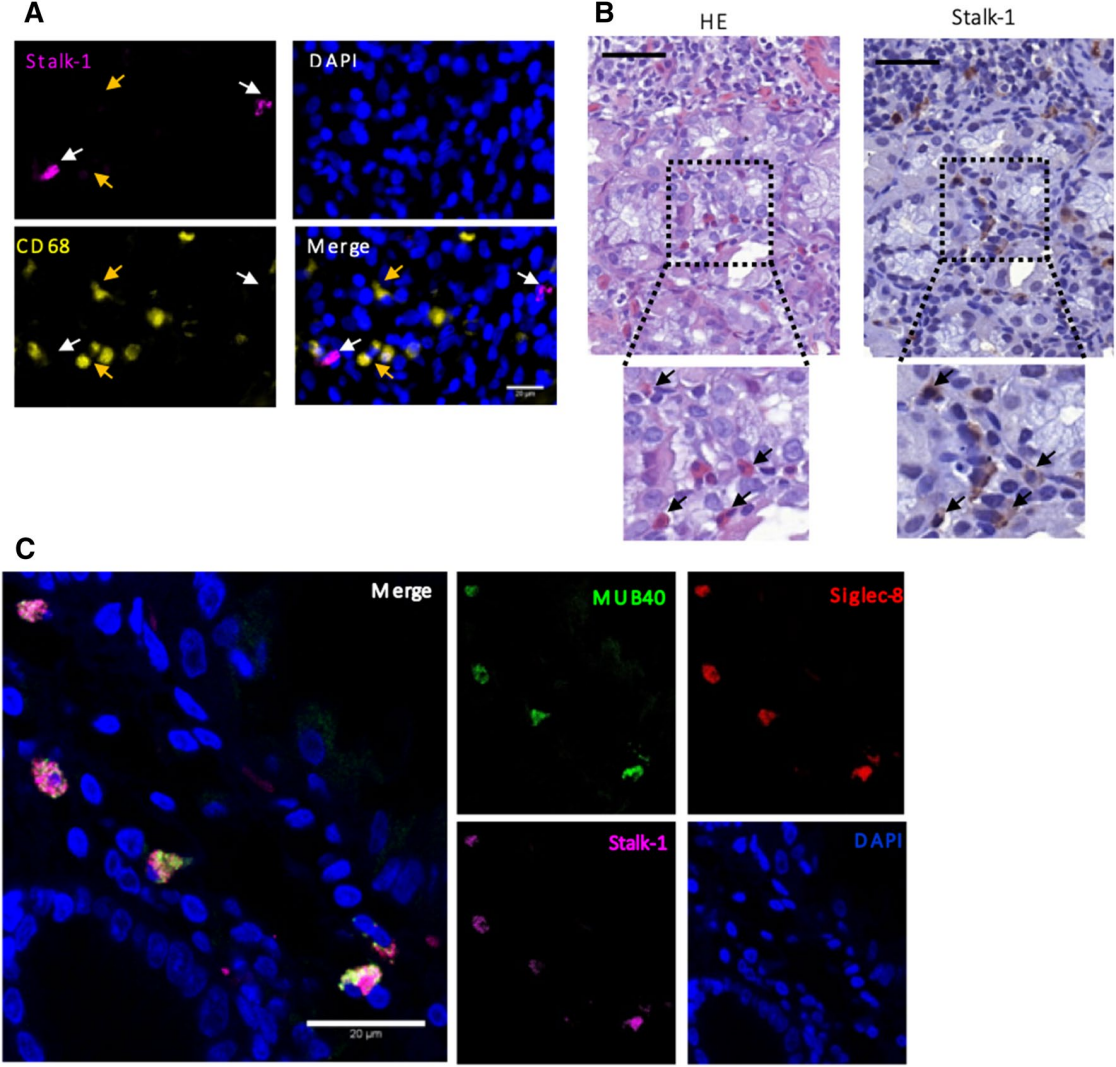
Identification of APRIL cytokine producing cells in gastric lymphoid infiltrates from patients with gastric MALT lymphoma. (**A**) Immunofluorescence co-labeling of the membrane form of APRIL with Stalk-1+ (magenta), CD68+ macrophages (yellow) and DAPI-labeled nuclei (blue) on sections of human gastric biopsies from GML patients. The white arrows indicate the APRIL-producing cells (Stalk-1+) and the yellow arrows the macrophages (CD68+). (**B**) Example of HE staining and APRIL staining (Stalk-1+) in serial sections of human gastric biopsies of GML patients. (**C**) Immunofluorescence co-labeling of the membrane form of the APRIL Stalk-1 (magenta), MUB40+/Siglec-8 + eosinophilic polynuclear cells (green and red) and DAPI-labeled nuclei (blue) on sections of human gastric biopsies of GML patients.

To identify the human producer cells of APRIL and its target cells, two antibodies were used for IHC experiments: Stalk-1 antibody and Aprily-2 antibody. The Stalk-1 antibody recognizes the non-secreted and membrane-bound form of the cytokine and thus the producing cells, whereas Aprily-2 recognizes the secreted and internalized form and thus the target cells^25^. The data obtained showed the presence of Stalk-1-positive cells within the infiltrates from GML patients (Fig. 4A, B). As suggested by Munari et al*.*^17^ we determined whether the cells producing APRIL were macrophages (CD68+) infiltrating the tumor. As immunofluorescence data did not show Stalk-1/CD68 co-labeling (Fig. 4A), we concluded that APRIL-producing cells within lymphoid infiltrates of GML patients were not macrophages, as previously described^17^. Hematoxylin and eosin staining (H&E) and IHC using Stalk-1 were performed on serial sections of FFPE tissue sections of the two gastric biopsies from GML patients. The APRIL-producer cells resembled polymorphonuclear leukocytes macroscopically and, in particular, eosinophils (Fig. 4B).

To validate this hypothesis, double immunofluorescence staining on purified human polymorphonuclear leukocytes was performed. The myelotracker (also known as MUB40-a neutrophil lactoferrin marker)^26,27^, coupled with cyanine-5 was used. Given the presence of granulation in the eosinophilic polynuclear cells, this compound could also mark this other type of polymorphonuclear leukocytes. Eosinophil-specific anti-Siglec-8 (sialic acid-binding immunoglobulin superfamily lectins) antibodies were used to stain human eosinophils^28^. This double MUB40-Siglec-8 staining was first validated on purified human eosinophilic polynuclear cells using normal human purified neutrophils as a control, after verifying the effectiveness of the purification by performing May-Grünwald Giemsa staining on the purified cells (Fig. S1A, B). As expected, the immunofluorescence results showed simple MUB40 + Siglec-8—labeling (Fig. S1C) for human neutrophils, whereas the human eosinophils were double labeled with MUB40 + Siglec-8 + (Fig. S1D). We analyzed human gastric biopsies of GML patients and found that Stalk1+ cells were double-positive for MUB40 and Siglec-8. In conclusion, within the lymphoid infiltrates, the cells producing APRIL appeared to be eosinophilic polynuclear cells (Fig. 4C).

### Target cells of the APRIL cytokine

**Figure 5:**
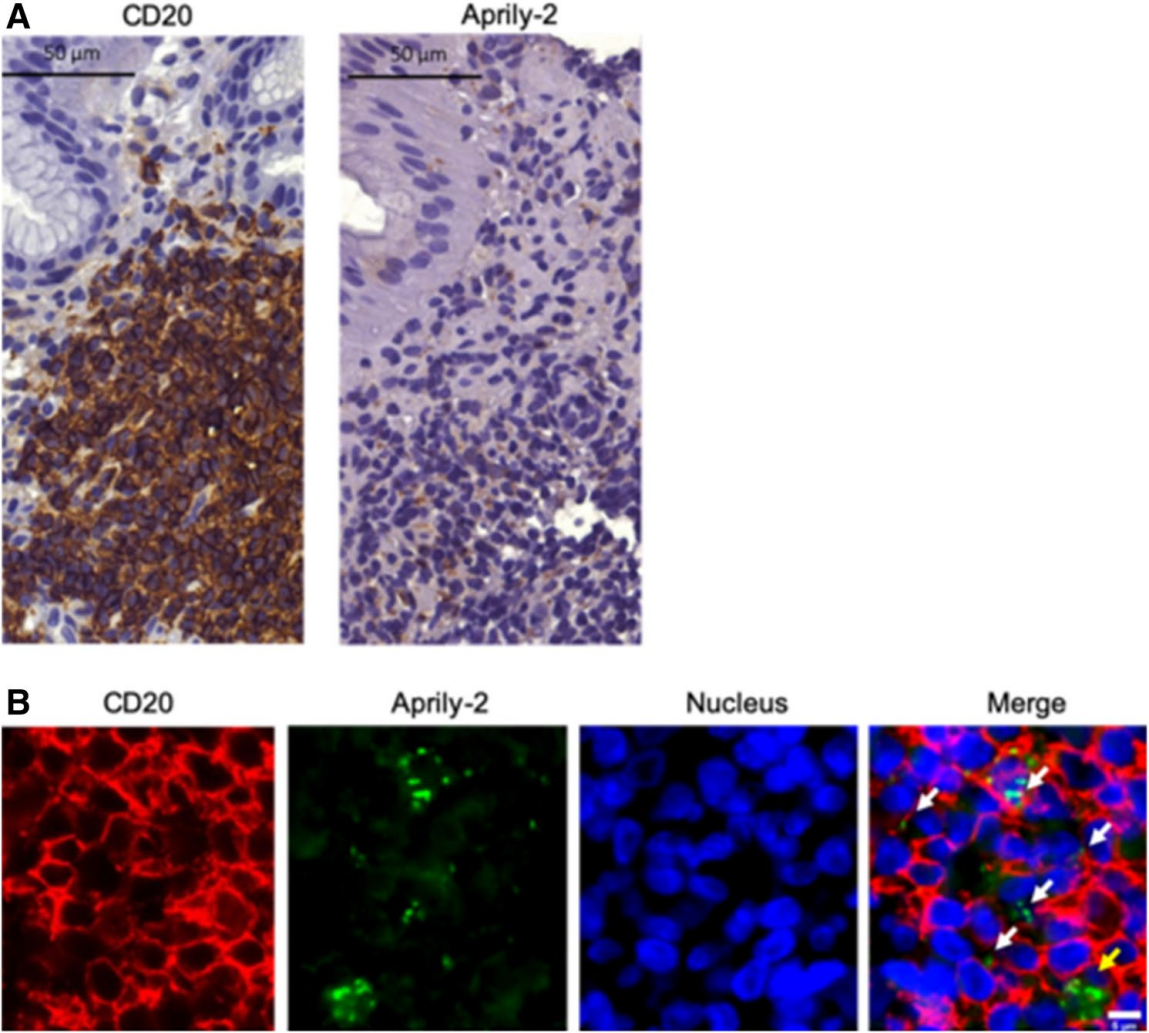
Identification of APRIL cytokine target cells in gastric biopsies from patients with gastric MALT lymphoma. (**A**) Example of B lymphocyte (CD20+) and APRIL (Aprily-2+) labeling in lymphoid infiltrates by IHC on sections of human gastric biopsies from GML patients. (**B**) Immunofluorescence co-labeling of B lymphocyte (CD20+ in red), APRIL cytokine (Aprily-2+ in green) and DAPI-labeled nuclei (blue) on sections of human gastric biopsies from GML patients. The white arrows indicate the B lymphocytes that internalized APRIL and the yellow arrows the CD20-cells that internalized the cytokine.

The presence of APRIL cytokine targeted cells with lymphoid infiltrates of human GML was estimated by IHC. Detection of the secreted and internalized form of the cytokine was performed on tissue sections of gastric biopsies from patients with GML, using the Aprily-2 antibody. Numerous cells present in the infiltrates were Aprily-2-positive (Fig. 5A). Co-labeling with Aprily-2 and CD20 was performed and analyzed by immunofluorescence (IF), revealing the co-localization of these two markers (white arrows, Fig. 5B). Therefore, the target cells of the APRIL cytokine were predominantly B lymphocytes. However, some cells did not exhibit Aprily-2/CD20 co-labeling (yellow arrows, Fig. 5B). As Munari et al.^17^ suggested that macrophages could be Aprily-2-positive, IF was performed on serial tissue sections of gastric biopsies from patients with GML using anti-CD68 and Aprily-2 antibodies (Fig. S2). Only rare CD68+/Aprily-2+ cells were found (orange arrow, Fig. S2), possibly corresponding to macrophages that phagocytized their environment and thus internalized APRIL.

In conclusion, the use of two distinct antibodies that differentiate APRIL producer cells from target cells, combined with appropriate cell markers, allowed us to demonstrate that tumor B cells in GML are targeted by this cytokine, which is produced by tumor-infiltrating eosinophilic polynuclear cells.

## Discussion

To understand more fully the pathophysiology of GML induced by *H. pylori* infection, an alternative model was implemented in our laboratory that relies on the use of transgenic C57BL6 mice for the human form of the APRIL cytokine (Tg-hAPRIL), and infected with *Helicobacter sp* (*H. pylori* or *H. felis*)^19^. We showed that the Tg-hAPRIL mouse model infected with *Helicobacter sp* closely reproduces the pathology of human GML with the same balanced gastric Th1, Th2, and Treg inflammatory response. The dysregulation of APRIL at the gastric level has been confirmed in patients with GML. B lymphocytes were identified as the target of the APRIL cytokine that appeared to be produced by tumor-infiltrating eosinophils.

We first investigated the local inflammatory response at the gastric level in murine models to identify the inflammatory environment favorable to the appearance of GML in infected Tg-hAPRIL mice. At the GML stage, the inflammatory response at the gastric level was balanced. Indeed, an increase in the expression of transcription factors for Th1 (*tbet*) and Th2 (*gata3*) responses was observed in both infected murine models (WT and Tg-hAPRIL mice). We also confirmed these findings in patients with GML. This balanced inflammatory response within the gastric microenvironment at GML stage was already described by D’Elios et al*.*^29^ who showed that gastric *H. pylori*-reactive T-cell clones from MALT lymphoma comprised a larger proportion of Th clones secreting both Th1- and Th2-type cytokines, suggesting that mechanisms other than cytokines could be involved in the *H. pylori*-dependent enhanced T-cell help for B-cell proliferation. The role of a regulatory response must also be considered. In infected Tg-hAPRIL mice, a more intense regulatory response was found in contrast to infected WT mice. The intensity of this response may explain the higher level of bacterial colonization in infected Tg-hAPRIL mice^27^ compared to infected WT mice. As suggested^5^, Treg cells could participate in the persistence of the pathogen in the gastric mucosa by delaying the inflammatory response to allow chronic antigen stimulation necessary for lymphoid proliferation.

The role of APRIL in cancers and lymphomas has been analyzed in several studies^12,14–16^.

Chonwerawong et al.^30^ have recently suggested that BAFF, another member of the TNF family, may be involved in the development of gastric mucosal hyperplasia and B-cell lymphoid follicles formation in mice lacking Nlrc5 (NLR family CARD domain-containing 5) infected by *H. felis*. Here, we highlight the deregulation of APRIL in patients with GML, compared to a control population of patients with gastritis, suggesting a potential role of this cytokine in the development of GML in humans. Indeed, we showed that tumor B cells internalize this cytokine. Since APRIL is involved in lymphocyte regulation and B lymphocyte development, overexpression of this cytokine could contribute to the significant proliferation of neoplastic B cells present in lymphoid infiltrates.

We also identified APRIL-producing cells in the lymphoid infiltrates of GML patients. To differentiate between the target cells and the cytokine producer cells, two antibodies were used: Stalk-1 and Aprily-2. The Stalk-1 antibody recognizes a peptide located at the level of the extracellular domain that remains associated with the membrane after proteolytic cleavage (aa 67–79, which correspond to the peptide GTGGPSQNGEGYP); it is therefore directed against the cells producing the cytokine. The Aprily-2 antibody is directed against the extracellular portion of the protein (aa 93–233) and the secreted form after proteolytic cleavage, thus allowing APRIL cytokine target cells to be recognized^25^. Our data show that APRIL was not produced by macrophages, unlike the report of Munari et al.^17^ which, in a different cohort of GML patients and using Aprily-2 antibody against the secreted form of APRIL, found APRIL production by macrophages (CD68^+^) infiltrating the tumor. The cells producing the APRIL cytokine within the tumor were identified in the present work as eosinophilic polynuclear cells. This identification was made possible by study of their cellular morphology (nuclear and cytoplasmic granulations) and the results of previously validated IF staining. The use of the myelotracker (MUB40) known as a neutrophil-specific marker allowed us to characterize Stalk-1 positive cells as polymorphonuclear leukocytes. The association with the anti-Siglec-8 antibody enabled us to distinguish between neutrophil (anti-Siglec-8 negative) and eosinophilic polynuclear cells (anti-Siglec-8 positive).

This distinction was important since neutrophil polynuclear cells were previously identified by Schwaller et al*.*^25^ as the major cellular source of the APRIL cytokine within tumor tissue infiltrates in patients with diffuse large B-cell lymphoma, which is part of non-Hodgkin B-cell lymphomas like GML. However, eosinophilic polynuclear cells were also identified as cells producing the APRIL cytokine in other studies^31–33^. Among the factors that allow the recruitment of these cells, exposure to pathogens such as *H. pylori* has already been reported^34^.

Eosinophils comprise part of the inflammatory reaction in *H. pylori* gastritis. Indeed, in 1991 McGovern et al.^35^ suggested that the severity of chronic gastritis was significantly correlated with eosinophil infiltration. A few years later, Aydemir et al.^36^ showed that eosinophil infiltration in gastric mucosa was greater in *H. pylori*-positive patients than in *H. pylori*-negative patients. Bold et al*.*^37^ also described the presence of eosinophils in the gastric lamina propria in 65% of *H. pylori*-positive chronic gastritis patients and the eosinophils were even more numerous in atrophic gastritis patients with intestinal metaplasia. It was therefore suggested that eosinophils may participate in the immune response against *H. pylori*. However, Arnold et al*.*^34^ recently showed that *H. pylori* escapes their bactericidal activity. As a result, we investigated the APRIL expression by eosinophils in gastritis and atrophic gastritis cases to see whether *H. pylori* infection could induce expression in vitro using eosinophils purified from peripheral blood of normal donors (coculture experiments). Unfortunately, neither *april* gene upregulation nor APRIL production were detected (data not shown), indicating that circulating eosinophils cells are probably different from tumor-infiltrating ones. This latter category could indeed be influenced by the tumor microenvironment and especially the local inflammatory response, which could not be reproduced in vitro.

Several studies have shown tumor-associated tissue eosinophilia in various types of tumors, such as in Hodgkin lymphoma where it is associated with poor prognosis^33^; however, to date, the presence of tumor-infiltrating eosinophils in the context of GML has been poorly described. Herrera-Goepfert et al*.*^38^ showed in a set of 35 low-grade and 16 high-grade GML patients that 73.8% exhibited an increased number of eosinophils accompanied by precancerous lesions such as intestinal metaplasia and atrophy. Piazuelo et al*.*^39^ proposed that eosinophils play a dual role in chronic gastritis by either downregulating or favoring the effects of Th1 proinflammatory cytokines. In the context of *H. pylori* infection, Arnold et al*.*^34^ showed in vivo that eosinophils suppress Th1 responses locally; this, in turn, could be an explanation for the bacterial gastric persistence, itself known to be involved in the initiation of the lymphomagenesis process. The identification of tumor-infiltrating eosinophils as the source of APRIL production in GML, as shown in the present study, is therefore of major interest. They no doubt play an important role in lymphocyte proliferation of targeted B-cells by their regulatory contribution^40^ to the inflammatory response and preservation of the bacterial load in vivo.

In conclusion, our results demonstrate that the cellular and inflammatory microenvironment is a key contributor to gastric lymphomagenesis. We now have an easy-to-implement model to evaluate the effect of different cytokine receptor inhibitors of the TNF family or activated signaling pathways on the establishment of GML. This is the first description of the implication of APRIL in low grade B cell lymphoma. In our opinion, the identification of APRIL production by eosinophils involved in GML paves the way for more extensive research on the pro-tumoral role of these cells. The targetability of the APRIL producing eosinophils in gastric MALT lymphomagenesis induced by *H. pylori* infection could represent an interesting strategy.

## Supplementary information

Supplementary Figure S1.

Supplementary Figure S2.

Supplementary Table S1.

Supplementary Legends.
