# Supplementary figures and images for: APRIL-producing eosinophils are involved in gastric MALT lymphomagenesis induced by Helicobacter sp infection
Source: Sci Rep. 2020 Sep 9;10:14858. doi: 10.1038/s41598-020-71792-3 (PMC7481773; doi:10.1038/s41598-020-71792-3)

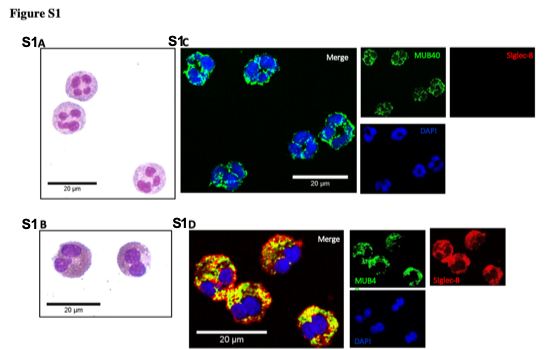

Supplement: Supplementary file 1 — Supplementary Figure S1. [file 41598_2020_71792_MOESM1_ESM.png]

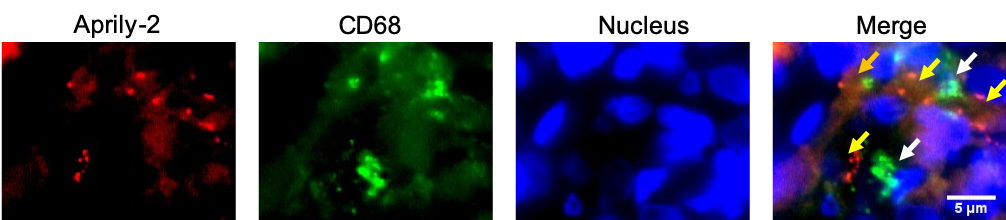

Supplement: Supplementary file 2 — Supplementary Figure S2. [file 41598_2020_71792_MOESM2_ESM.jpg]
