# Supplementary material for: APRIL-producing eosinophils are involved in gastric MALT lymphomagenesis induced by Helicobacter sp infection
Source: Sci Rep. 2020 Sep 9;10:14858. doi: 10.1038/s41598-020-71792-3 (PMC7481773; doi:10.1038/s41598-020-71792-3)
Supplement: Supplementary file 3 — Supplementary Table S1. [file 41598_2020_71792_MOESM3_ESM.docx]

Eosinophil-APRIL producing cells are involved in gastric MALT lymphomagenesis induced by *Helicobacter sp* infection.

Blosse Alice, Peru Sara, Levy Michael, Marteyn Benoit, Floch Pauline, Sifré Elodie, Giese Alban, Carlotti Martina, Azzi Martin Lamia, Dubus Pierre, Mégraud Francis, Ruskone Fourmestraux Agnès, Fabiani Bettina, Copie-Bergman Christiane, Robe Cyrielle, Hahne Michael, Huard Bertrand, Lehours Philippe.

**Table S1**. Antibodies and peptides used in the present study.
