# Supplementary material for: APRIL-producing eosinophils are involved in gastric MALT lymphomagenesis induced by Helicobacter sp infection
Source: Sci Rep. 2020 Sep 9;10:14858. doi: 10.1038/s41598-020-71792-3 (PMC7481773; doi:10.1038/s41598-020-71792-3)
Supplement: Supplementary file 4 — Supplementary Legends. [file 41598_2020_71792_MOESM4_ESM.docx]

**Supplementary Figure 1:** Co-labeling of purified human neutrophils and eosinophils. (S1A) May-Grünwald Giemsa staining of purified human neutrophils and purified human eosinophilic polynuclear cells; (S1B) Immunofluorescence co-labeling MUB40 (green), Siglec-8 (red) and DAPI (blue) of purified human neutrophil polynuclear cells (MUB40 + Siglec-8-); (S1C) purified human eosinophilic polynuclear cells MUB40 + Siglec-8 + (S1D).

**Supplementary Figure 2:** Immunofluorescence co-labeling of macrophages (CD68+ in green), APRIL cytokine (Aprily-2 + in red) and DAPI-labeled nuclei (in blue) on sections of human gastric biopsies from GML patients. The white arrows indicate the CD68+/Aprily-2- cells, the yellow arrows the CD68-/Aprily-2+ cells, and the orange arrow the double positive cells.
